# A high-resolution dataset for future compound hot-dry events under climate change

**DOI:** 10.1038/s41597-024-03883-z

**Published:** 2024-09-27

**Authors:** Yizhuo Wen, Junhong Guo, Feng Wang, Zhenda Hao, Yifan Fei, Aili Yang, Yurui Fan, Faith Ka Shun Chan

**Affiliations:** 1 Key Laboratory of Environmental Biotechnology, Xiamen University of Technology, Xiamen, 361024 China; 2 School of Geographical Sciences, University of Nottingham Ningbo China, Ningbo, 315100 China; 3 MOE Key Laboratory of Resource and Environmental, System Optimization, College of Environmental Science and Engineering, North China Electric Power University, Beijing, 102206 China; 4 State Key Laboratory of Earth Surface Processes and Resource Ecology, Faculty of Geographical Science, Beijing Normal University, Beijing, 100875 China; 5 Sustainability Standards Research Center, School of Economics, Central University of Finance and Economics, Beijing, 100081 China; 6 Department of Civil and Environmental Engineering, Brunel University London, Uxbridge, London, UB8 3PH Middlesex UK; 7 Water@Leeds and School of Geography, University of Leeds, Leeds, LS2 9JT UK

**Keywords:** Projection and prediction, Natural hazards

## Abstract

Global climate change is leading to an increase in compound hot-dry events, significantly impacting human habitats. Analysing the causes and effects of these events requires precise data, yet most meteorological data focus on variables rather than extremes, which hinders relevant research. A daily compound hot-dry events (CHDEs) dataset was developed from 1980 to 2100 under various socioeconomic scenarios, using the latest NASA Earth Exchange Global Daily Downscaled Projections (NEX-GDDP-CMIP6) dataset to address this. The dataset has a spatial resolution of 0.25 degrees (approximately 30 kilometres), including three indicators, namely D (the yearly sum of hot-dry extreme days), prI (the intensity of daily precipitation), and tasI (the intensity of daily temperature). To validate the accuracy of the dataset, we compared observational data from China (National Meteorological Information Center, NMIC), Europe (ERA5), and North America (ERA5). Results show close alignment with estimated values from the observational daily dataset, both temporally and spatially. The predictive interval (PI) pass rates for the CHDEs dataset exhibit notably high values. For a 90% PI, D has a pass rate exceeding 85%, whilst prI and tasI respectively show a pass rate above 70% and 95%. These results underscore its suitability for conducting global and regional studies about compound hot-dry events.

## Background & Summary

Global climate change contributes to an increasing trend in compound extreme events, resulting in widespread adverse impacts on the environment and humanity^1,2^. The IPCC AR6 report highlights that, as global warming intensifies, the likelihood of compound events occurring in many regions is set to rise^3^. Under the influence of climate change, compound hot-dry events (CHDEs) are projected to become notably more frequent^4^. Existing research indicates an anticipated increase in CHDEs across Europe, Asia, North America and Australia^5–8^. Consequently, the risk of heightened occurrences of CHDEs is anticipated in the future^9^. Regions prone to frequent wildfires, such as Australia, may experience an elevation in wildfire incidents due to the increased frequency of compound hot-dry events^10,11^. That becomes imperative to enhance future assessments of changes in compound hot-dry events, along with research into driving factors and their resultant impacts^12^.

Currently, research studies on CDHEs are being conducted worldwide, comparing the characteristics, driving factors, variations (detection, attribution, and prediction), and impacts of compound dry-hot conditions. Observational findings indicate an overall increase in regional and global CDHEs over the past few decades^13^. Experimental results, coupling the Weather Research and Forecasting (WRF) model with urban canopy parameterisation, demonstrate that climate warming induced by greenhouse gases is a primary driver for the increased frequency and duration of CDHE event^14^. Studies on impacts suggest that by the end of the 21st century, depending on different scenarios, an additional 700 million to 1.7 billion people globally will be exposed to expanding compound events. Furthermore, the cultivated land affected by these events is expected to increase by 2-5.7 million square kilometers^9^. These studies underscore the importance of effective governance in mitigating and managing the escalating risks associated with compound events.

A comprehensive understanding of causative analysis and impact assessment relies heavily on a substantial and dependable dataset, which significantly advances related research^15,16^. Hydrological datasets play a pivotal role in facilitating regional water resource management^17^. Global Climate Model (GCM) and Regional Climate Model (RCM) datasets supplement observational gaps, addressing research voids in regions with data insufficiency^18^. Despite the introduction of the advanced CMIP6 dataset, which provides high-precision climate data, prevailing meteorological data repositories predominantly offer variables-based data rather than event-based data. This divergence complicates the research landscape, amplifying the workload associated with relevant investigations^19,20^.

The absence of comprehensive compound event datasets may stem from divergent definitions employed in various studies. The IPCC categorises compound events as instances where multiple extreme events occur simultaneously, sequentially, or in distinct regions^21^. Consequently, researchers often define compound events based on the specific impacts they investigate within their studies^22,23^.

The objective of this paper is to facilitate research focused on the impacts of CHDEs (Compound Hot-Dry Events). To achieve this, we propose a universally recognised definition for CHDEs. In this definition, extreme events of mean temperature and precipitation are characterised as values above the 90th percentile and below the 10th percentile, respectively, which have gained widespread acceptance and served as the prevailing method for studying CHDEs^12,24–27^. Using this established definition, we aim to construct a comprehensive global dataset that encompasses both the intensity and duration of CHDEs. This dataset will be instrumental in capturing the spatiotemporal characteristics of CHDEs, providing valuable support for future research endeavours in this field.

## Methods

### Data source

Compound hot-dry events were calculated based on the NASA Earth Exchange Global Daily Downscaled Projections (NEX-GDDP-CMIP6) gridded dataset^28^. The NEX-GDDP-CMIP6 dataset comprises a total of 25 models. In our study, we have selected the 11 most widely utilised models (as shown in Table 1), which is deemed sufficient to establish the stability of the results.

**Table 1:** CMIP6 models used in this study.

Model name	Modelling center	Variant
ACCESS-CM2	Commonwealth Scientific and Industrial Research Organization	r1i1p1f1
ACCESS-ESM1-5	Commonwealth Scientific and Industrial Research Organization	r1i1p1f1
BCC-CSM2-MR	Beijing Climate Center, China Meteorological Administration	r1i1p1f1
CanESM5	Commonwealth Scientific and Industrial Research Organization	r1i1p1f1
CNRM-ESM2-1	Centre National de Recherches Météorologiques/ Centre Européen de Recherche et Formation Avancée en Calcul Scientifique	r1i1p1f2
INM-CM4-8	Institute for Numerical Mathematics	r1i1p1f1
IPSL-CM6A-LR	Institute Pierre Simon Laplace	r1i1p1f1
KACE-1-0-G	Korean Air Quality Model for Climate and Health Effects	r1i1p1f1
MIROC6	Atmosphere and Ocean Research Institute, The University of Tokyo and Japan Agency for Marine-Earth Science and Technology	r1i1p1f1
MPI-ESM1-2-HR	Max Planck Institute for Meteorology	r1i1p1f1
UKESM1-0-LL	Max Planck Institute for Meteorology	r1i1p1f2

Observed daily mean temperature and precipitation data were obtained from the National Meteorological Information Center (CN0.5, http://data.cma.cn) and fifth generation ECMWF (European Centre for Medium-Range Weather Forecasts) atmospheric reanalysis of the global climate (ERA5, https://cds.climate.copernicus.eu/cdsapp). The CN0.5 dataset has a high degree of correlation with the original sequence and small errors, making it an accurate reflection of spatiotemporal precipitation and temperature characteristics^29^. ERA5, based on global observational data, numerical models, and physical parameterisation schemes, employs data assimilation and numerical simulation techniques to reconstruct and simulate weather conditions over the past several decades (from 1940 to the present). This process generates high spatiotemporal resolution atmospheric and surface variable data. The data can be utilised in various fields such as climate research, weather analysis, climate model validation, environmental monitoring, and more^30^.

### Compound hot-dry events

Our analysis primarily focuses on the average temperature and precipitation during the warm season (in terrestrial regions), specifically the three consecutive months with the highest average temperature. For each model, extreme events for mean temperature and precipitation are respectively defined as values exceeding the 90th percentile and falling below the 10th percentile of the distribution obtained from each GCM member’s data over the 1981-2010 period (thereby defining extreme events in the warm climate based on historical percentile thresholds). Subsequently, utilising historical period thresholds, the assessment of forthcoming CHDEs is conducted across various Shared Socioeconomic Pathways (SSPs).

In detail, on a global scale, we independently model 265,272 grid points. Initially, we organize daily evaluation temperature and precipitation data for each grid during the historical period (1981-2010), sorting them in ascending order. We then calculate the 90th percentile for average temperature and the 10th percentile for average precipitation, which serve as the thresholds for CHDEs. When a specific day satisfies both criteria, we identify it as a compound hot and dry event occurrence at that point.

To further analyse temporal and spatial characteristics of CHDEs, we defined the frequency (i.e., D), precipitation intensity (i.e., prI) and temperature intensity (i.e., tasI) as follows:

1 $$D=\mathop{\sum }\limits_{i=1}^{N}{d}_{i}$$

2 $$prI=\frac{\mathop{\sum }\limits_{i=1}^{N}\frac{{\sum }_{j=1}^{{d}_{i}}(p{r}_{i,j}-p{r}_{thres,j})}{{d}_{i}}}{N}$$

3 $$tasI=\frac{\mathop{\sum }\limits_{i=1}^{N}\frac{{\sum }_{j=1}^{{d}_{i}}(ta{s}_{i,j}-ta{s}_{thres,j})}{{d}_{i}}}{N}$$

where N is the number of extreme events within a particular, D is the yearly sum of hot-dry extreme days, i is the subscript of a hot-dry event, and j is the day in the hot-dry event (i). d_i_ is the duration of a hot-dry event (i), *pr*_i,j_ and *tas*_i,j_ is the daily precipitation and temperature at the day j during the hot-dry extreme event (i), *pr*_*thres,j*_ and *tas*_*thres,j*_ are the 10^th^ percentile threshold for the daily precipitation and 90^th^ percentile threshold for the daily temperature at the day *j*. The definitions of frequency and intensity of CHDEs were developed here following the relevant definitions of compound heatwaves proposed in Ma and Yuan^31^. These indices enable us to identify and quantify CHDEs effectively across diverse geographical locations and serve as a basis for our research analysis.

## Data Records

Our dataset can be accessed from the associated permanent DOI (10.6084/m9.figshare.24038790.v6)^32^. Each site encompasses four SSP-RCPs (SSP1-2.6, SSP2-4.5, SSP3-7.0, SSP5-8.5) and three distinct periods: historical (1981-2010), 2050 s (2041-2070), and 2080 s (2071-2100). Each scenario encompasses three variables at a monthly time step: duration (D), precipitation intensity (prI), and temperature intensity (tasI). The dataset has a spatial resolution of 0.25 degrees (approximately 30 kilometres). We have organised the data for each scenario into netcdf files with the data information for each index of CHDEs provided in Table 2.

**Table 2:** Data information for each index of CHDEs in the netcdf files.

Index	duration (D)	temperature intensity (tasI)	precipitation intensity (prI)
Dimensions	longitude = 1400	longitude = 1400	longitude = 1400
	latitude = 600	latitude = 600	latitude = 600
	time = unlimited//30 currently	time = unlimited// 30 currently	time = unlimited// 30 currently
Variables	double longitude (longitude)	double longitude (longitude)	double longitude (longitude)
	longitude: units = “degrees_east”	longitude: units = “degrees_east”	longitude: units = “degrees_east”
	longitude: long_name = “longitude”	longitude: long_name = “longitude”	longitude: long_name = “longitude”
	double latitude (latitude)	double latitude (latitude)	double latitude (latitude)
	latitude: units = “degrees_north”	latitude: units = “degrees_north”	latitude: units = “degrees_north”
	latitude: long_name = “latitude”	latitude: long_name = “latitude”	latitude: long_name = “latitude”
	int time (time)	int time (time)	int time (time)
	time: units = “years since 0”	time: units = “years since 0”	time: units = “years since 0”
	time: long_name = “time”	time: long_name = “time”	time: long_name = “time”
	time: calender = “standard”	time: calender = “standard”	time: calender = “standard”
	double Total_Duration(time, latitude, longitude)	double Tas_Intensity(time, latitude, longitude)	double Pr_Duration(time, latitude, longitude)
	Total_Duration: units = “day”	Tas_Intensity: units = “K”	Pr_Duration: units = “kg/m^2/s”
	Total_Duration: _FillValue = −9999	Tas_Intensity: _FillValue = −9999	Pr_Duration: _FillValue = −9999

## Technical Validation

**Fig. 1:**
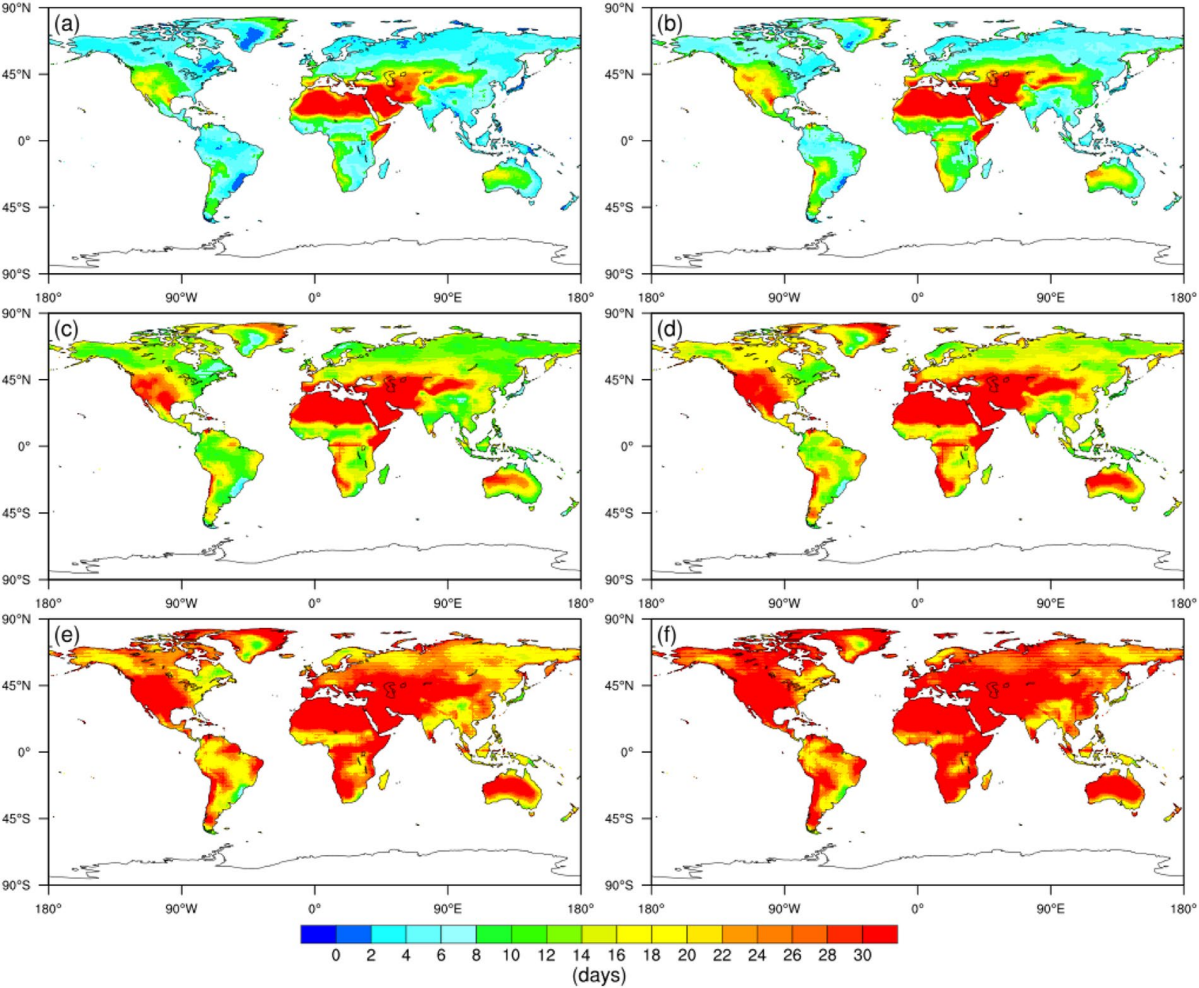
Future duration of CHDEs (i.e., D) under the SSP2-4.5 scenario for the 2050 s (left column) and 2080 s (right column): (**a**,**b**) 10th percentile, (**c**,**d**) 50th percentile and (**e**,**f**) 90th percentile.

Figure 1 illustrates the future projected annual mean frequency of compound extreme hot and dry events under the SSP2-4.5 scenario, considering different percentile levels. Spatially, it is expected that in the future, there will be an increased concentration of compound hot and dry events between 0° and 45° north latitude at the 50th percentile level, particularly in western Asia, North America, and southern Africa. The southern regions of Greenland and central Australia are also likely to experience longer durations of dry and hot compound events amidst global warming. Temporally, the areas impacted by compound events are anticipated to expand during the 2070 s compared to the 2050 s.

**Fig. 2:**
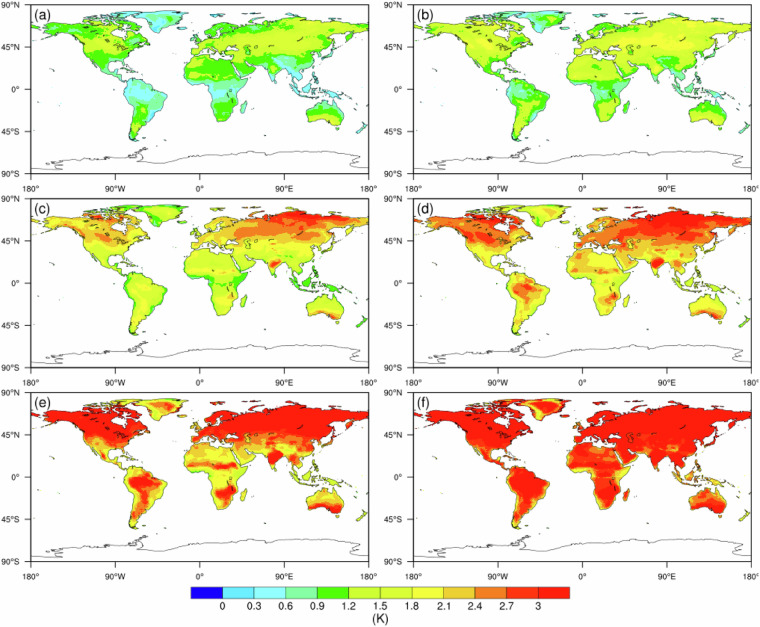
Future temperature intensity (i.e., tasI) of extreme hot events under the SSP2-4.5 scenario for the 2050 s (left column) and 2080 s (right column): (**a**,**b**) 10th percentile, (**c**,**d**) 50th percentile and (**e**,**f**) 90th percentile.

**Fig. 3:**
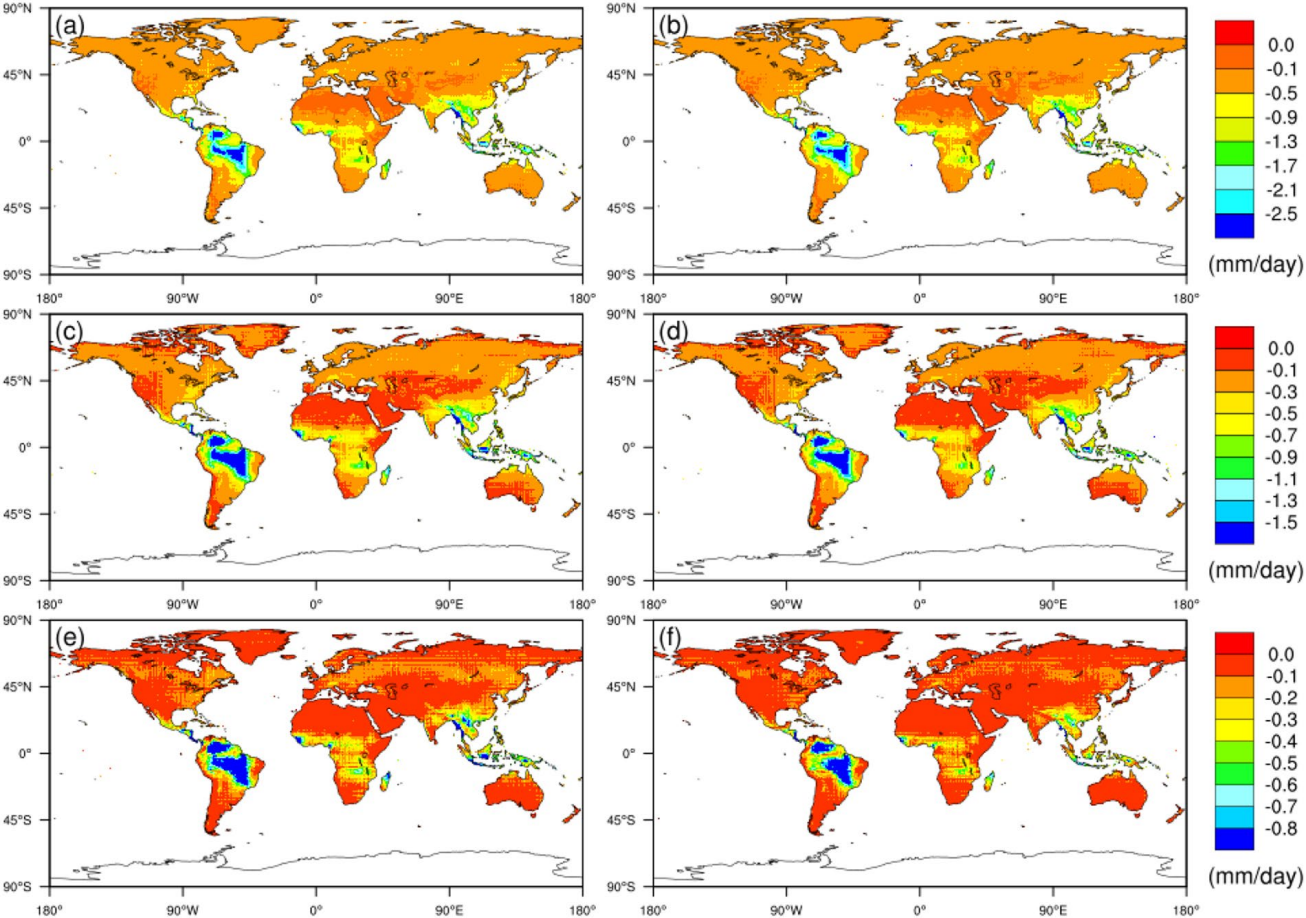
Future precipitation intensity (i.e., prI) of extreme dry events under the SSP2-4.5 scenario for the 2050 s (left column) and 2080 s (right column): (**a**,**b**) 10th percentile, (**c**,**d**) 50th percentile and (**e**,**f**) 90th percentile.

Figures 2 and 3 display the spatial distribution of hot-dry extreme event intensity under the SSP2-4.5 scenario. The intensity of these compound events is measured by two indicators: the deviation of daily mean temperature from the historical 90th percentile value (i.e., *tasI*), and the deviation of daily precipitation from the historical 10th percentile baseline (i.e., *prI*). Regarding temperature, there is a clear spatial pattern where the Northern Hemisphere exhibits higher intensity compared to the southern regions. At the end of this century, it is expected that larger areas will encounter more intense extreme heat events. For precipitation, compared to the threshold in the baseline (10th percentile), the corresponding precipitation deficit is predominantly observed near or south of the equator, particularly in most parts of South America, indicating that the intensity of drought in this region will worsen. In other words, these regions will experience a significantly higher severity of drought events compared to other areas.

The accuracy of CHDEs relies significantly on the reliability of the meteorological variables used for their computation. The CN0.5 dataset has been widely utilised and validated. Similarly, we employed this dataset to identify data for the three variables during the historical period. Using China, North America and Europe as examples, we performed a quality control check on the dataset output by comparing results from 14,752 calculations with values extracted from CN0.5. Simultaneously, we applied the same methodology to compare the North American Region (47,964) and the European Region (18,844). All these were done to ensure that the numerical outputs fall within realistic ranges.

Table 3 illustrates the probabilities of the calculated mean variables falling within the range of observed data results. While values exceeding these ranges are not necessarily erroneous, they should not occur frequently. In fact, within a 100% range, these disparate values only reached a rate of 0.29% (D), 1.48% (prI) and 0% (tasI) in China. In both Europe and North America, the differences are similarly minimal. The specific figures are as follows: in Europe, they are 0%, 0%, and 0.42%, while in North America, they are 0.01%, 0.19%, and 2.81%. Within a 90% range, the pass rates are also quite satisfactory, with a pass rate of 85.21% for D, 86.59% for prI, and 98.09% for tasI in China. For Europe, the pass rates are 100.00% for D, 78.43% for prI and 99.17% for tasI. In North America, the figures are 99.99% for D, 71.31 for prI and 95.50% for tasI. These results indicate a favorable level of consistency between the calculated values and observed data, reinforcing the validity and reliability of our approach in assessing CHDEs.

**Table 3:** Comparison of Pass Rates.

	Range	Parameter		
		Duration	prI	tasI
China	90% PI Pass Rate	85.21%	86.59%	98.09%
	100% PI Pass Rate	99.71%	98.52%	100.00%
Europe	90% PI Pass Rate	100.00%	78.43%	99.17%
	100% PI Pass Rate	100.00%	100.00%	99.58%
North America	90% PI Pass Rate	99.99%	71.31%	95.50%
	100% PI Pass Rate	99.99%	99.81%	97.19%

^*^PI: predictive interval.

**Fig. 4:**
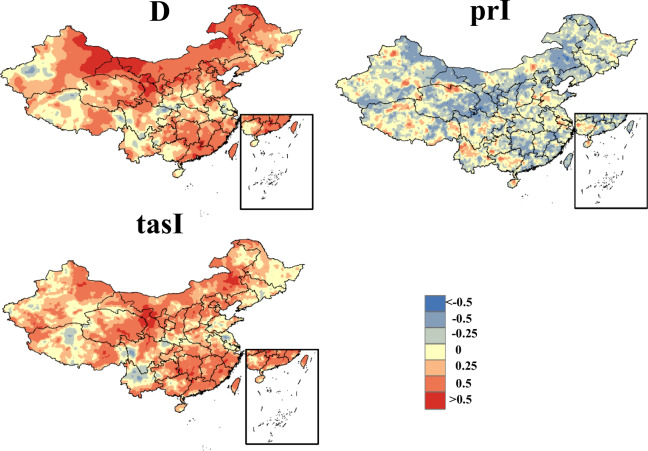
Spatial patterns covering China Pearson correlation coefficient between CHDEs dataset and CN0.5 during a 30-year period (1981-2010).

**Fig. 5:**
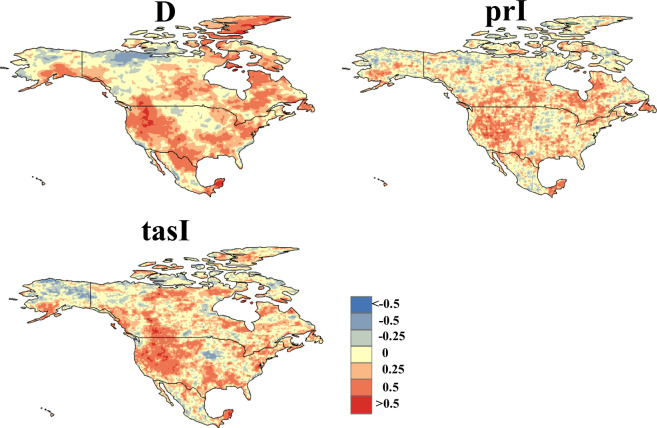
Spatial patterns covering the North America Pearson correlation coefficient between CHDEs dataset and ERA5 during 30 years (1981-2010).

Furthermore, we assessed the spatial correlation of the data, revealing a strong consistency between the duration and temperature variables across both CHDEs dataset and CN0.5 datasets (as shown in Fig. 4). The Pearson correlation coefficient for the precipitation variable is not as robust as the first two variables, but this is not unexpected due to the inherent challenges in precipitation simulation at a daily temporal and spatial resolution. However, given high pass rates for the 90% predictive interval shown in Table 3, the precipitation intensity (i.e., prI) in the observed CHDEs can be likely bracketed by the predictive intervals in the CHDE dataset from 11 GCMs. These findings reinforce the reliability of our data assessment process, indicating that despite the challenges in accurately estimating daily precipitation at high spatial or temporal resolutions, the overall results are robust and dependable Fig. 5.

**Fig. 6:**
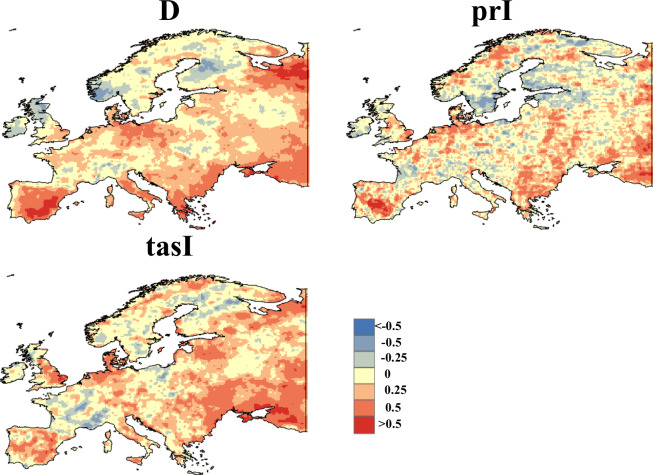
Spatial patterns covering Europe Pearson correlation coefficient between CHDEs dataset and ERA5 during 30 years (1981-2010).

**Fig. 7:**
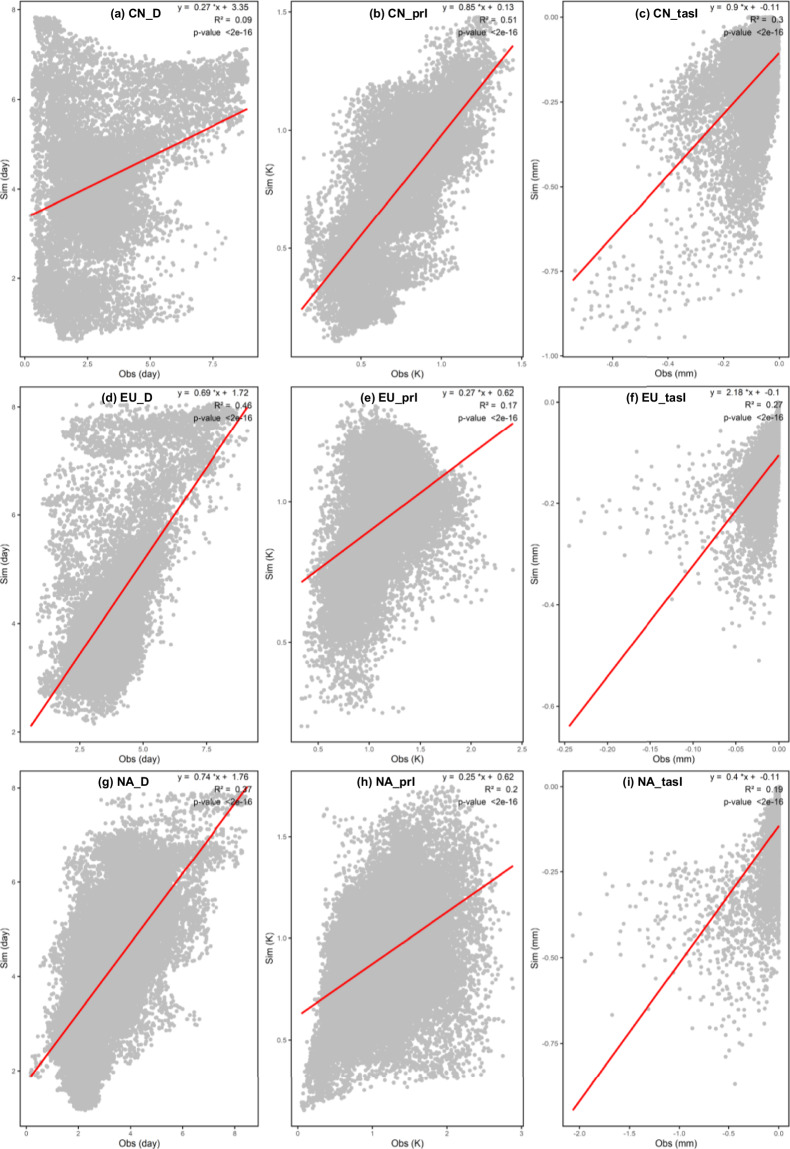
Spatial correlation between CHDEs from GCM simulations and those from observations in China (CN), Europe (EU) and North America (NA).

Similarly, we compared the CHDEs dataset and data from ERA5 to present a more equitable evaluation. The comparison methods were consistent, as illustrated in Figs. 6 and 7. From the figures, it can be observed that the results for Europe and North America are roughly similar to those for China. In fact, for the parameter prI, we anticipate that the results for Europe and North America would be superior to those for China, possibly due to differences in the observational datasets. This divergence may arise because ERA5 data undergo reconstruction and simulation, while CN0.5 data undergoes only spatial interpolation, resulting in relatively lower correlation. Moreover, we calculated the spatial correlation between the mean values of D, prI, and tasI and the corresponding observations over the three tested region. The results indicate significant correlation between CHDE indices (i.e., D, prI, tasI) and those from observations with P-values less than 0.04. (as shown in Fig. 7). In summary, akin to the findings in the Chinese region, the validation of the CHDEs dataset in Europe and North America is considered reliable. This dataset can be utilised for subsequent impact assessments and other analyses in these regions.

## Limitations

Similarly, our study has some limitations. Specifically, due to computational and data storage constraints, we selected 11 CMIP6 datasets developed by renowned research institutions worldwide instead of utilising the complete set of 25 models. There are also certain shortcomings in terms of uncertainty, and future research will seek more collaborations to meet hardware requirements and reduce uncertainty arising from the number of models. Regarding validation, we only validated the data for three regions: China, Europe, and North America. The experimental results confirmed the reliability of the CHDEs dataset, researchers in other Regions, particularly those with higher accuracy requirements, are advised to conduct their validation when using this data. We will continue our efforts to extend validation to more countries and regions.
