# Author Correction: A high-resolution dataset for future compound hot-dry events under climate change

**DOI:** 10.1038/s41597-024-04054-w

**Published:** 2024-11-05

**Authors:** Yizhuo Wen, Junhong Guo, Feng Wang, Zhenda Hao, Yifan Fei, Aili Yang, Yurui Fan, Faith Ka Shun Chan

**Affiliations:** 1 Key Laboratory of Environmental Biotechnology, Xiamen University of Technology, Xiamen, 361024 China; 2 School of Geographical Sciences, University of Nottingham Ningbo China, Ningbo, 315100 China; 3 MOE Key Laboratory of Resource and Environmental, System Optimization, College of Environmental Science and Engineering, North China Electric Power University, Beijing, 102206 China; 4 State Key Laboratory of Earth Surface Processes and Resource Ecology, Faculty of Geographical Science, Beijing Normal University, Beijing, 100875 China; 5 Sustainability Standards Research Center, School of Economics, Central University of Finance and Economics, Beijing, 100081 China; 6 Department of Civil and Environmental Engineering, Brunel University London, Uxbridge, London, UB8 3PH Middlesex UK; 7 Water@Leeds and School of Geography, University of Leeds, Leeds, LS2 9JT UK

Correction to: *Scientific Data* 10.1038/s41597-024-03883-z, published online 27 September 2024

In this article the author’s name Yizhuo Wen was incorrectly written as Yizhou Wen. The original article has been corrected.

